# The nutritional value of beef from Polish Red and Limousin cattle breeds maintained by an extensive production system

**DOI:** 10.5194/aab-67-259-2024

**Published:** 2024-06-21

**Authors:** Konrad Wiśniewski, Marcin Świątek, Jolanta Król, Beata Kuczyńska

**Affiliations:** 1 Institute of Animal Sciences, Warsaw University of Life Sciences, Ciszewskiego 8, 02-786 Warsaw, Poland; 2 Institute of Quality Assessment and Processing of Animal Products, University of Life Sciences in Lublin, Akademicka 13, 20-950 Lublin, Poland

## Abstract

The aim of the study was to compare the quality of beef in three separate types of muscles – musculus longissimus thoracis, musculus infraspinatus, and musculus longissimus lumborum – obtained from the carcasses of bulls of a local breed of Polish Red and of two lines of Limousin maintained by an extensive production system using year-round pasture and bale grazing. Studies of the lumbar longissimus muscle showed that the Limousin breed (LM FR) was characterised by a higher protein content and the lowest intramuscular fat content. The carnosine content showed significant differences between the types of muscles and breeds; however, the anserine content showed minimal differences. The research showed separate characteristics in terms of the meat composition parameters of Limousin and Polish Red bulls. The beef of the Limousin breed had favourable nutritional value, higher protein content, and reduced fat content, potentially contributing to increased tenderness. The beef of local-breed cattle – although, in every respect, not equal to specialised meat breeds – showed features suitable for the production of meat with unique nutritional value, including a favourable essential fatty acid profile, especially with a higher conjugated linoleic acid (CLA) content.

## 1 Introduction

The primary goal of breeding and raising beef cattle is to produce animals that yield meat that matches consumer preferences through efficient feeding and management practices (Litwińczuk et al., 2006, 2016; Biel et al., 2019; Presumido et al., 2020; Madhusankha and Thilakarathna, 2021; Araújo et al., 2022; Kuczyńska, 2022; Mansfield et al., 2023). To optimise the quality of meat obtained from meat breeds, it is important to use appropriate fattening and maintenance systems such as feeding on pastures throughout the year. Environmental factors, including the maintenance system (extensive, semi-intensive, and intensive), significantly influence the proper growth and rearing of bulls. In Poland, the predominant practice involves fattening calves from dairy breeds, notably the Polish Holstein-Friesian breed, and crossbreeds from commercial crossbreeding in dairy herds (Momot et al., 2020; Solarczyk et al., 2020). The predominant breed of beef cattle is the Limousin, currently constituting 75.8 % of the population, though this is still increasing (Kostusiak et al., 2023). This breed originated in the northwestern part of the Massif Central in France, specifically from the Limousin and Marche regions (Kostusiak et al., 2019). This breed evolved from two wild ancestors: the big-horned aurochs (*Bos primigenius*) and the short-horned aurochs (*Bos brachyceros*). The homeland of this breed comprises regions with a harsh climate and rocky soil. Undoubtedly, these challenging environmental conditions have influenced features such as the animals' health, fitness, and adaptability. These animals thrive in both livestock-building intensive breeding and in extensive breeding on pastures. There are numerous reasons for the immense popularity of Limousin cattle. In addition to their impressive musculature and the high culinary value of their lean meat, the ability to adapt to various environmental conditions, their good health, their ease of calving, and the good survival rate of the calves contribute to their appeal. On the other hand, Limousin cattle, a French breed, are known for their lean and muscular body. Limousin beef is often praised for its tenderness and low fat content, making it the preferred choice for lean cuts of meat. However, this means Polish native breeds are gradually being replaced by modern varieties that produce more milk and achieve greater weight gain.

Local breeds include Polish Red cattle, which have been covered by the genetic resources protection programme in Poland since 1999. The Polish Red cattle breed is one of the four native cattle breeds in Poland and is derived from brachyceric short-horned cattle. As of December 2022 (data from the National Research Institute of Animal Production in Balice), the population of Polish Red cattle in Poland is 4108 animals and has been gradually increasing since 2010. Currently, Polish Red cattle constitute 1 % of the domestic animal population and only 0.29 % of the registered population. Due to the relatively low milk yield of Polish Red cows, which is only 3520 kg, meat production is a real alternative for the preservation of this breed (Wiśniewski and Kuczyńska, 2022). The breed is characterised by an excellent ability to adapt to difficult environmental conditions prevailing not only in foothill and mountain areas but also in Warmia and Mazury in Poland. They demonstrate an effective use of agricultural feed, especially dry loose feed in winter and pasture grass in summer; are undemanding in the selection of feed; are resistant to diseases; and demonstrate extreme longevity. The excellent meat composition and culinary values of this breed, its rich chemical composition, and the improving functional properties of the meat confirm its name as a typical meat breed. The Polish Red and Limousin cattle breeds have distinct characteristics. Polish Red cattle, originating from Poland, are known for their endurance and adaptability. Generally, the meat of Polish Red cattle is valued for its excellent taste, tenderness, and distinct marbling, which may be an indicator of its high quality. These features can be attributed to year-round grazing on pastures rich in plant diversity and high outdoor activity (Korzekwa et al., 2023).

As with any breed, factors such as diet and care significantly influence the quality of beef (Litwińczuk et al., 2016; Wiśniewski et al., 2021). The appropriate selection of culinary cuts and the types of muscles present in them are also important (Litwińczuk et al., 2006; Wiśniewski et al., 2021; Hammond et al., 2022). Beef contains a substantial number of biologically active proteins, which are crucial for various physiological functions, encompassing antioxidant and antiinflammatory responses and neurological, muscular, retinal, immune, and cardiovascular activities. Notably, taurine, carnosine, anserine, and L-carnitine play vital roles. The consumption of 30 g of dried beef can fulfil an adult's daily physiological requirement for these bioproteins. Carnosine ( β -alanyl-L-histidine), a dipeptide discovered in cattle, and anserine ( β -alanyl-1-methyl-L-histidine), its methylated product, are protein components that aid human immune defences against infections caused by bacteria; fungi; parasites; and viruses, including corona viruses. They strengthen the metabolism and functions of immune system cells such as monocytes and macrophages, positively impacting metabolic, immunological, retinal, muscular, cartilage, neurological, and cardiovascular health (Bagirov et al., 2014; Radzik-Rant et al., 2020; Wu, 2020). Beef fat contains biologically active lipid compounds of significant functional importance, including essential fatty acids (EFAs), conjugated linoleic acid (CLA) dienes, lipophilic vitamins, and phospholipids. These compounds possess antioxidant properties, which inhibit the formation of carbonyl groups in proteins. Proteins also exhibit chelating and antiglycation properties. Essential fatty acids, which the body cannot produce independently, serve as primary precursors for omega-3 and omega-6 fatty acids. All these components contribute positively to human health (Troy et al., 2016; Choi et al., 2000).

The many factors affecting the quality of beef include diet (French et al., 2001; Randby et al., 2010), the animal production system (Duckett et al., 2009; Pighin et al., 2016), the genotype and age of slaughter (Florek et al., 2007; Guzek et al., 2013; Momot et al., 2020), cooking technique (Rant et al., 2019), and the specific cut of meat (Jeremiah et al., 2003; Hammond et al., 2022). Different breeds have unique characteristics that influence factors such as tenderness, flavour, and fat distribution in the meat (Christensen et al., 2011; Liu et al., 2022). Understanding these diverse elements is crucial when evaluating and comparing beef from different sources, highlighting the need for comprehensive information to empower consumers to make informed choices in line with their preferences. The aim of the study was to compare the quality of beef in three separate types of muscles – musculus longissimus thoracis, musculus infraspinatus and musculus longissimus lumborum – obtained from the carcasses of bulls of a local breed of Polish Red and of two lines of Limousin maintained by an extensive production system using year-round pasture and bale grazing

## 2 Materials and methods

### 2.1 Animal and rearing conditions

The research was conducted on two farms in Poland that specialised in beef cattle breeding and that featured Polish Red (PRC) and Limousin cattle breeds. Rigorous measures were taken to select animals with minimal relations, aiming to encompass the full spectrum of genetic diversity within each breed. Both lines were maintained on the same farm, under the same environmental conditions. This is the result of years of breeding work conducted on the farm. The breeds differed in terms of carcass weight and daily weight gain.

The Limousin breed was chosen for comparison in this study because it is the most popular breed in Poland. This decision is likely based on its widespread prevalence in the region, its adaptation to local conditions, and its market demand and on potential research focusing on specific breed characteristics. The study aims to reflect the practical and economic aspects of the local agricultural context, and the choice of the Limousin breed aligns with these considerations.

To standardise the impact of management and breeding systems on meat quality, a uniform beef management system was employed for all breeds, which were representative of the selected farms. This included a standardised diet over 10 months, with animals grazing on pasture and receiving a specified diet. All animals were maintained by an extensive production system using year-round pasture and, additionally, bale grazing in winter season feeding. The pasture sward included meadow fescue (40 %), perennial ryegrass (30 %), alfalfa (15 %), and white clover (15 %). In the winter season feeding, a satisfactory diet was bale grazing, consisting of grass silage (approx. 60 %) and meadow hay (approx. 30 %). During the finishing period (4 months before slaughter), bulls were fattened via the addition of extra concentrate (approx. 10 %), straw and barley added to sodium bicarbonate (0.6 %), and appropriate vitamin supplements (1.5 %), all fed ad libitum.

Calves were kept with their mothers in the pastures until weaned, utilising a bale-grazing feeding system or enriched undersown annual plants, following the free-range method. Animals had constant access to water and salt licks. The initial body weights of bulls were 233±5 kg for Polish Red cattle and 281±3 kg for Limousin cattle. Intact bulls born in autumn were selected for slaughter, with 55 being from the PRC herd, 21 being from the Limousin herd of French origin (LM FR), and 32 being from the Limousin herd of Polish origin (LM PL). The bulls had a 100 % breed genotype and were transported to the nearest slaughter point at a distance of 150±30 km from the farms.

On the day of slaughter, the bulls exhibited the following characteristics:

- ages from 24 months for the Limousin breed to 30 months for Polish Red cattle
- body weights of 570±20 kg for Polish Red cattle and 666±40 kg for Limousin cattle
- carcass weights of 350±30 kg for Polish Red cattle and 440±27 kg for Limousin cattle
- daily weight gains of approximately 900 g for Polish Red cattle and 1166 g for Limousin.

Carcasses were processed according to standard protocols without the use of electrical stimulation, maintaining the carcass cooling temperature as per the International Committee for Animal Recording (ICAR) requirements throughout the European Union (ICAR, 2018). Post-slaughter, the carcasses were aged at 4 °C for 5 d, and three cuts were obtained from each carcass: musculus infraspinatus from the shoulder, musculus longissimus thoracis from the rib-eye, and musculus longissimus lumborum from the roast-beef section.

### 2.2 Meat sample preparation

The research material comprised 324 beef muscle samples from PRC, LM FR, and LM PL obtained after slaughter. The muscles selected were the musculus infraspinatus (cut between the 6th and 13th ribs, equivalent to the culinary cut known as the shoulder), musculus longissimus thoracis (culinary equivalent of a cut called entrecote with rib-eye), and musculus longissimus lumborum (culinary equivalent of a cut known as roast beef), all harvested from the left carcass. Each sample, weighing approximately 300 g, was vacuum packed and frozen at -18 °C in preparation for chemical analyses. Muscle samples were intact before packaging, and no subcutaneous fat or connective tissue were removed. One professional butcher cut up all the carcasses. A portion of steak was cut from each logging site in the identic location, and it came from the same central logging site for each killed bull, maintaining a consistent anatomical location for each animal.

These meat samples, representative of each group, were transported to the research laboratory of the Animal Breeding Department at the Institute of Animal Sciences in Warsaw.

### 2.3 Chemical analysis

The beef samples underwent chopping, followed by placement in a blender and grinding until homogeneity was achieved. The basic chemical composition of the raw meat was determined by analysing moisture, crude protein, and intramuscular fat using a Food Scan™ analyser. This near-infrared spectroscopy (NIRS) technique employed transmission within the 850–1050 nm range and artificial neural networks, following the method described by Wiśniewski et al. (2021).

Meat fat extraction was conducted using the Folch method (AOAC, 1990). Fatty acid methylation was carried out in accordance with the EN ISO 55093 (ISO, 2000) transesterification method. The functional fatty acid content was determined using an Agilent 7890A GC gas chromatograph with a flame ionisation detector, HP Chem Station software, and a VARIAN Select FAME column (length: 100 m, diameter: 0.25 mm, layer thickness: 0.25 µ m; Perlan Technologies, Waldbronn, Germany), as per Kuczyńska et al. (2015). Each peak was identified using pure methyl ester standards, and the fatty acids were identified using reference material BCR 163 (beef and pig fat blend). The linoleic acid (CLA) isomer was determined using the standard *cis*-9, *trans*-11 octadecadienoic acid (Supelco).

The measurement of the antioxidant dipeptides anserine and carnosine was conducted using an RP-HPLC (reverse-phase high-performance liquid chromatograph) Agilent 1100 instrument and a Jupiter 5u C18 300A column, following the methodology outlined by Łukasiewicz et al. (2018).

Based on the content of selected fatty acids and the sum of selected fatty acid groups, atherogenic indices (AIs) and thrombogenic indices (TIs) were calculated using equations developed by Ulbricht and Southgate (1991).

### 2.4 Statistical analysis

Meat attributes were evaluated through an analysis of variance using ANOVA in IBM SPSS Statistics 29.0 software. For each variable, the mean (LSM) and standard error of the mean (SEM) were calculated. In cases where statistically significant differences were observed, Tukey's test was conducted, with the statistical significance set at p≤0.05 . The level of significance was determined after performing a preliminary statistical analysis.

## 3 Results

### 3.1 Differences in the content of protein and intramuscular fat and related indicators of health-promoting features in three types of muscles obtained from Limousin and Polish Red bulls

The protein contents in the longissimus thoracis show significant differences between breeds. Limousin of French origin (LM FR) demonstrated greater protein content (24.16 %) by approximately 11.59 % compared to Polish Red cattle (PRC), which had a protein content of 21.65 %. Similarly, Limousin of Polish origin (LM PL) had a higher content (24.10 %) by about 11.31 % compared to PRC. There were fewer differences, relatively, for the infraspinatus. LM FR exhibited a slightly higher content (22.23 %) by approximately 0.63 % compared to PRC (22.09 %), while LM PL exhibited a lower content (22.03 %) by about 0.27 % compared to PRC. The most pronounced differences were observed for the longissimus lumborum. LM FR demonstrated a substantially greater content (24.20 %) by approximately 15.24 % compared to PRC (21.00 %), while LM PL had a higher content (23.67 %) by about 12.71 % compared to PRC. In summary, the percentage differences between the groups highlight the variability in protein content across the different muscles and breeds (Table 1).

**Table 1:** The effect of breed on protein content (%) in three types of muscles in Limousin and Polish Red cattle. The percentage (%) variation in relation to PRC is shown in parentheses.

Breed	M. longissimus thoracis	M. infraspinatus	M. longissimus lumborum
PRC	21.65 ${}^{A,B}$	22.09	21.00 ${}^{A,B}$
LM FR	24.16 ${}^{A}$ ( +11.59% )	22.23 ( +0.63% )	24.20 ${}^{A}$ ( +15.24% )
LM PL	24.10 ${}^{B}$ ( +11.31% )	22.03 ( -0.27% )	23.67 ${}^{B}$ ( +12.71% )
SEM	0.163	0.074	0.152
p value	0.010	0.075	0.010

PRC – Polish Red cattle, LM FR – Limousin of French origin, LM PL – Limousin of Polish origin. Means (in columns) marked with the same letters differ significantly for uppercase letters at p≤0.01 . SEM – standard error of the mean.

The intramuscular fat content in the longissimus thoracis revealed substantial differences between breeds. Limousin of French origin (LM FR) had a content (2.02 %) that was notably lower, by approximately 63.93 %, than that of Polish Red cattle (PRC), which had a fat content of 5.60 %. Similarly, Limousin of Polish origin (LM PL) had a significantly lower content (1.92 %) by about 65.72 % compared to PRC. In the infraspinatus, the differences in intramuscular fat content were relatively small. LM FR had a slightly lower content (1.96 %) by approximately 1.01 % compared to PRC (1.98 %), while the LM PLs' content (2.01 %) was not practically different but was greater by about 1.51 % compared to PRC. The most significant differences in intramuscular fat content were observed in the longissimus lumborum. LM FR exhibited a substantially smaller content (1.79 %) by approximately 56.97 % compared to PRC (4.16 %), while LM PL showed a significantly lower content (2.79 %) by about 32.93 % compared to PRC. In summary, the percentage differences highlight the relative changes in intramuscular fat content between the different breeds and muscles (Table 2).

**Table 2:** The effect of breed on the intramuscular fat content (%) in three types of muscles in Limousin and Polish Red cattle. The percentage (%) variation in relation to PRC is shown in parentheses.

Breed	M. longissimus thoracis	M. infraspinatus	M. longissimus lumborum
PRC	5.60 ${}^{A,B}$	1.98	4.16 ${}^{A,B}$
LM FR	2.02 ${}^{A}$ ( -63.93% )	1.96 ( -1.01% )	1.79 ${}^{A}$ ( -56.97% )
LM PL	1.92 ${}^{B}$ ( -65.72% )	2.01 ( + 1.51 %)	2.79 ${}^{B}$ ( -32.93% )
SEM	0.273	0.041	0.274
p value	0.010	0.082	0.010

PRC – Polish Red cattle, LM FR – Limousin of French origin, LM PL – Limousin of Polish origin. Means (in columns) marked with the same letters differ significantly for uppercase letters at p≤0.01 . SEM – standard error of the mean.

The anserine content in the longissimus thoracis shows modest differences between breeds. LM FR exhibited a slightly higher content (74.30 mg 100 g ${}^{-1}$ ) by approximately 1.54 % compared to Polish PRC, which had an anserine content of 73.17 mg 100 g ${}^{-1}$ . On the other hand, LM PL had a slightly lower content (72.00 mg 100 g ${}^{-1})$ by about 1.61 % compared to PRC. For the infraspinatus, the differences in anserine content were relatively small. LM FR had a content that was not practically different but still greater (74.09 mg 100 g ${}^{-1}$ ), by approximately 0.94 % compared to PRC (73.40 mg 100 g ${}^{-1}$ ), while LM PL had negligibly lower content (73.28 mg 100 g ${}^{-1}$ ) by about 0.16 % compared to PRC. The differences in anserine content were minimal in the longissimus lumborum. LM FR had a content that was not practically different but still greater (74.92 mg 100 g ${}^{-1}$ ) by approximately 0.94 % compared to PRC (74.22 mg 100 g ${}^{-1}$ ), while LM PL had no significant difference (0.00 %) compared to PRC. In summary, the percentage differences highlight the relative changes in anserine content between the different breeds and muscles (Table 3).

**Table 3:** The effect of breed on the anserine content (mg 100 g ${}^{-1}$ of meat) in three types of muscles in Limousin and Polish Red cattle. The percentage (%) variation in relation to PRC are shown in parentheses.

	M. longissimus thoracis	M. infraspinatus	M. longissimus lumborum
PRC	73.17	73.40	74.22
LM FR	74.30 ( +1.54% )	74.09 ( +0.94% )	74.92 ( +0.94% )
LM PL	72.00 ( -1.60% )	73.28 ( -0.99% )	74.22 (0 %)
SEM	0.931	0.384	0.368
p value	0.068	0.062	0.079

PRC – Polish Red cattle, LM FR – Limousin of French origin, LM PL – Limousin of Polish origin, SEM – Standard error of the mean.

The cattle breed had a significant impact on the carnosine content in the tested muscle types (Table 4). LM FR exhibited a significantly greater content (451.61 mg 100 g ${}^{-1}$ ), by approximately 30.38 %, than PRC, which had a carnosine content of 346.52 mg 100 g ${}^{-1}$ . Conversely, LM PL had a notably smaller content (322.03 mg 100 g ${}^{-1})$ , by about 7.06 %, than PRC. For the infraspinatus, the differences in carnosine content were notable. LM FR had significantly greater content (324.05 mg 100 g ${}^{-1})$ , by approximately 10.41 %, than PRC (293.44 mg 100 g ${}^{-1})$ ; while LM PL had a considerably smaller content (271.94 mg 100 g ${}^{-1})$ , by about 7.34 %, than PRC. The differences in carnosine content were pronounced in the longissimus lumborum. LM FR exhibited a substantially smaller content (274.24 mg 100 g ${}^{-1})$ , by approximately 12.44 %, than PRC (313.15 mg 100 g ${}^{-1})$ ; while LM PL had significantly less content (254.53 mg 100 g ${}^{-1})$ , by about 18.75 %, than PRC. In summary, the percentage differences highlight the relative changes in carnosine content between the different breeds and muscles.

**Table 4:** The effect of breed on the carnosine content (mg 100 g ${}^{-1}$ of meat) in three types of muscles in Limousin and Polish Red cattle. The percentage (%) variation in relation to PRC are shown in parentheses.

Breed	M. longissimus thoracis	M. infraspinatus	M. longissimus lumborum
PRC	346.52 ${}^{a}$	293.44 ${}^{a}$	313.15 ${}^{a,b}$
LM FR	451.61 ${}^{a,b}$ ( +30.33% )	324.05 ${}^{a,b}$ ( +10.43% )	274.24 ${}^{a}$ ( -12.42% )
LM PL	322.03 ${}^{b}$ ( -7.06% )	271.94 ${}^{b}$ ( -7.32% )	254.53 ${}^{b}$ ( -18.72% )
SEM	17.778	1.100	1.150
p value	0.050	0.050	0.050

PRC – Polish Red cattle, LM FR – Limousin of French origin, LM PL – Limousin of Polish origin. Means (in columns) marked with the same letters differ significantly for lowercase letters at p≤0.05 . SEM – standard error of the mean.

The highest contents of the C18:2 *cis*-9, *trans*-11 CLA isomer in beef were obtained from local Polish Red bulls and ranged from 10.14 to 10.70 g 100 g ${}^{-1}$ of meat. CLA in longissimus thoracis indicates slight differences between the origins of the breeds. Limousin showed a similar, slightly lower content (ranging from 9.19 to 9.30 mg 100 g ${}^{-1}$ ) than PRC, the content of which was 10.1 mg 100 g ${}^{-1}$ . Similarly, LM PL was characterised by a much lower content (9.30 mg 100 g ${}^{-1}$ ) by approximately 8.28 % compared to PRC. In the case of the infraspinatus muscle, differences in CLA content were relatively small. LM FR had a slightly lower content (10.08 mg 100 g ${}^{-1}$ ) by approximately 1.75 % compared to PRC (10.26 mg 100 g ${}^{-1}$ ), while LM PL had a practically unchanged lower content (10.04 mg 100 g ${}^{-1}$ ) by approximately 2.14 % compared to PRC. Differences in CLA content were noticeable in the lumbar longissimus muscle. LM FR was characterised by a significantly higher content (10.31 mg 100 g ${}^{-1}$ ) by approximately 3.64 % compared to PRC (10.70 mg 100 g ${}^{-1}$ ), while LM PL had a much lower content (9.91 mg 100 g ${}^{-1}$ ) by approximately 7.38 % compared to PRC (Table 5).

**Table 5:** The effect of breed on the C18:2 *cis*-9, *trans*-11 CLA content (mg 100 g ${}^{-1}$ of meat) in three types of muscles in Limousin and Polish Red cattle. The percentage (%) variation in relation to PRC is shown in parentheses.

Breed	M. longissimus thoracis	M. infraspinatus	M. longissimus lumborum
PRC	10.14 ${}^{a,b}$	10.26	10.70
LM FR	9.19 ${}^{a}$ ( -9.36% )	10.08 ( -1.75% )	10.31 ( -3.64% )
LM PL	9.30 ${}^{b}$ ( -8.28% )	10.04 ( -2.14% )	9.91 ( -7.38% )
SEM	0.132	0.135	0.218
p value	0.050	0.082	0.059

PRC – Polish Red cattle, LM FR – Limousin of French origin, LM PL – Limousin of Polish origin. Means (in columns) marked with the same letters differ significantly for lowercase letters at p≤0.05 .

The omega-6 / omega-3 ratio in the longissimus thoracis reveals substantial differences between breeds. LM FR exhibited a significantly higher ratio (4.590) (approximately 1.33 times higher) than Polish Red cattle (PRC), which had a ratio of 3.445. Similarly, LM PL, with a ratio of 4.258, was about 1.24 times higher than PRC. For the infraspinatus, the differences in the omega-6 / omega-3 ratio were relatively small. LM FR, with a value of 3.692, was approximately 1.03 times higher than the PRC ratio, which was 3.585. Meanwhile, LM PL, with a value of 3.547, had a slightly lower ratio, approximately 0.99 times that of PRC. The differences in the omega-6 / omega-3 ratio were noticeable in the longissimus lumborum. LM FR, with a ratio of 2.390, was substantially lower, approximately 0.67 times lower than that of PRC (3.550). Similarly, LM PL, with a ratio of 2.730, was lower at about 0.77 times that of PRC (Table 6).

**Table 6:** The effect of breed on the omega-6 / omega-3 ratio in three types of muscles in Limousin and Polish Red cattle. The percentage (%) variation in relation to PRC is shown in parentheses.

Breed	M. longissimus thoracis	M. infraspinatus	M. longissimus lumborum
PRC	3.445	3.585	3.550 ${}^{A,B}$
LM FR	4.590 ( +33.23% )	3.692 ( +2.98% )	2.390 ${}^{A}$ ( -32.67% )
LM PL	4.258 ( +23.59% )	3.547 ( -1.06% )	2.730 ${}^{B}$ ( -23.94% )
SEM	0.109	0.051	0.113
p value	0.055	0.075	0.010

PRC – Polish Red cattle, LM FR – Limousin of French origin, LM PL – Limousin of Polish origin. Means (in columns) marked with the same letters differ significantly for uppercase letters at p≤0.01 . SEM – standard error of the mean.

The atherogenic index for the longissimus thoracis shows subtle differences between breeds. LM FR had a slightly lower atherogenic index of 0.726 compared to PRC, which had an index of 0.741. Similarly, LM PL also exhibited a slightly lower atherogenic index at 0.738. For the infraspinatus, the differences in the atherogenic index were minor. LM FR had a slightly higher atherogenic index of 0.738 compared to PRC, which had an index of 0.733. On the other hand, LM PL showed a slightly lower atherogenic index of 0.729. The atherogenic index varied noticeably in the longissimus lumborum. LM FR exhibited a higher atherogenic index of 0.764 compared to PRC, which had an index of 0.741. Conversely, LM PL had a slightly lower atherogenic index of 0.737. In summary, the atherogenicity index reflects the potential cardiovascular risk associated with the fatty acid composition of meat. These subtle differences between muscle types are important in countering negative attitudes toward red-meat consumption, especially in the context of cardiovascular health. Lower values in terms of the atherogenicity index are generally considered to be more beneficial for the health of potential beef consumers (Table 7).

**Table 7:** The effect of breed on the atherogenic index in three types of muscles in Limousin and Polish Red cattle. The percentage (%) variation in relation to PRC is shown in parentheses.

Breed	M. longissimus thoracis	M. infraspinatus	M. longissimus lumborum
PRC	0.741	0.733	0.741
LM FR	0.726 ( -2.02% )	0.738 ( +0.68% )	0.764 ( +3.10% )
LM PL	0.738 ( -0.40% )	0.729 ( -0.54% )	0.737 ( +0.80% )
SEM	0.004	0.003	0.003
p value	0.069	0.073	0.081

PRC – Polish Red cattle, LM FR – Limousin of French origin, LM PL – Limousin of Polish origin, SEM – standard error of the mean.

The thrombogenic index for the longissimus thoracis reveals subtle differences between breeds. LM FR had a slightly lower thrombogenic index of 1.521 compared to PRC, which had an index of 1.564. Similarly, LM PL also exhibited a slightly lower thrombogenic index at 1.543. For the infraspinatus, the differences in the thrombogenic index were minor. LM FR had a slightly higher thrombogenic index of 1.485 compared to 1.478 for PRC. By contrast, LM PL showed a slightly lower thrombogenic index of 1.475. The thrombogenic index varied noticeably in the longissimus lumborum. LM FR exhibited a higher thrombogenic index of 1.648 compared to the PRC index of 1.545. Conversely, LM PL had a slightly lower thrombogenic index of 1.556 (Table 8).

**Table 8:** The effect of breed on the thrombogenic index in three types of muscles in Limousin and Polish Red cattle. The percentage (%) variation in relation to PRC is shown in parentheses.

Breed	M. longissimus thoracis	M. infraspinatus	M. longissimus lumborum
PRC	1.564	1.478	1.545
LM FR	1.521 ( -2.75% )	1.485 ( +0.47% )	1.648 ( +6.66% )
LM PL	1.543 ( -1.34% )	1.475 ( -0.20% )	1.556 ( +0.71% )
SEM	0.009	0.007	0.011
p value	0.085	0.082	0.072

PRC – Polish Red cattle, LM FR – Limousin of French origin, LM PL – Limousin of Polish origin, SEM – standard error of the mean.

## 4 Discussion

Studies have shown that animal breeds (including native breeds) (Litwińczuk et al., 2016; Kim et al., 2020) and muscles type (Jeremiah et al., 2003; Florek et al., 2007) have an impact on meat taste and composition. Ultimately, the best way to determine what type of beef consumers prefer is for them to try both and decide based on their own taste and culinary preferences (Araújo et al., 2022). Additionally, the specific maintenance, diet, and handling of animals may vary from farm to farm; thus, it is important to consider these factors as well when assessing beef quality (Pighin et al., 2006; Presumido et al., 2020; Korzekwa et al., 2023). For example, the protein content of beef can vary depending on factors such as the age of the animal (Litwińczuk et al., 2006), the cut of meat (Hammond et al., 2022), and the animal's diet (Purchas and Zou, 2008; Bagirov et al., 2014; Puente et al., 2019; Solarczyk et al., 2020; Wiśniewski et al., 2021; Terry et al., 2021).

PRC exhibited a lower body weight (570 kg) compared to both of the Limousin breeds: 680 kg for LM FR and 652 kg for LM PL. This difference may influence carcass weight and has implications for overall meat yield. Carcass weight is a crucial metric for assessing the economic value of the slaughtered animal (Kim et al., 2020). PRC had a lower carcass weight (350 kg) compared to the Limousin breeds (450 kg for LM FR and 430 kg for LM PL), indicating differences in the meat yield per animal. Daily weight gain is an important parameter reflecting the efficiency of feeding and management practices (Momot et al., 2020; Terry et al., 2021). Both Limousin breeds demonstrated higher daily weight gains (1176.50 g for LM FR and 1154.98 g for LM PL) compared to PRC (900.54 g). This suggests that Limousin breeds achieve faster growth rates during the finishing period. The differences in body weight and daily weight gain suggest that Limousin breeds may have advantages in terms of meat production efficiency.

The substantial differences in protein content between the Limousin breeds (LM FR and LM PL) and PRC indicate inherent disparities that are influenced by genetic factors (Wiśniewski et al., 2021). The LM FR consistently demonstrated higher protein content across all three muscles compared to both PRC and LM PL. The study emphasises that the extent of the differences in protein content varies across different muscles. For instance, the longissimus lumborum exhibited more pronounced differences compared to the infraspinatus. Limousin cattle are known for producing lean beef with a relatively high protein content. Our research has shown that LM meat is characterised by a higher protein percentage for all analysed muscles. The highest protein content was found in the M. lumborum of the LM FR breed (24.20 mg 100 g ${}^{-1}$ ), but, for this muscle, this was 3 percentage points lower than in the meat of the native breed. These results are comparable to other studies (Wiśniewski et al., 2021; Kostusiak et al., 2023; Kuczyńska, 2022). The muscles in a beef carcass can be divided into two main types: working muscles and supporting muscles. For example, the muscles of the hind legs and fore legs (working muscles) tend to have a higher protein content compared to the muscles of the ribs and lumborum (supporting muscles).

The study underscores substantial differences in intramuscular fat content between the Limousin breeds (LM FR and LM PL) and PRC. LM FR exhibits a remarkably low intramuscular fat content compared to both PRC and LM PL. These findings suggest inherent breed-specific disparities in fat deposition. Intramuscular fat, commonly referred to as marbling, plays a crucial role in meat tenderness and flavour (Troy et al., 2016). The observed decrease in intramuscular fat content, especially in the longissimus lumborum of LM FR, suggests potential implications for the tenderness and flavour profile of the meat (Jeremiah et al., 2003; Puente et al., 2019). Understanding the intramuscular fat differences between breeds and muscles has practical implications for meat producers. It can inform breeding programmes, feeding strategies, and marketing approaches so that they align with consumer demands for specific fat levels in beef (Puente et al., 2019; Bermingham et al., 2021; Terry et al., 2021). Intramuscular fat content that is good for meat quality should be within 2 %–3 % (Kostusiak et al., 2023). In French cattle breeds, selection for muscle mass has been shown to be associated with reduced intramuscular fat. For example, the main meat breeds, Limousin, Charolaise, and Blonde Aquitaine, have less intramuscular fat than Salers breeds reared under the same conditions (Christensen et al., 2011).

Carnosine and anserine are recognised as biologically active meat proteins (Wu, 2020). There was a significant increase in protein content, especially in LM FR and the longissimus lumborum, confirmed for only the anserine content in this muscle and breed. The carnosine content in the longissimus lumborum in LM FR and LM PL was significantly less than that of PRC, indicating relative changes in carnosine content between the studied LM breeds. The amount of carnosine in meat, due to its connection with oxygen metabolism, may also depend on the type of muscle and the proportions of the individual fibres it contains. Muscles with a higher content of oxidative and oxidative–glycolytic fibres may have a higher carnosine content. Oxidative red muscles, characterised by low glycogen levels but high lipid concentrations, may involve carnosine in energy production (Radzik-Rant et al., 2020; Wu, 2020). This may explain the higher content of this compound in the infraspinatus in the conducted research. In this study, the genotype had no effect on the content of the analysed bioactive protein components in beef muscles, while for the Limousin breeds of both origins and for Polish Red cattle, there was no effect of muscle type on the anserine content. Similarly, Purchas et al. (2004) found no differences in the values of this parameter between muscles when examining the longissimus and sub-spiral muscles of fresh beef. In a study by Kostusiak et al. (2023), the LM longissimus dorsi muscle was characterised by a significantly higher content in terms of both anserine and carnosine.

Beef and other ruminant products are important dietary sources of CLA (conjugated linoleic acid), especially the *cis*-9, *trans*-11 isomer, which has been identified to be a crucial health-promoting factor that has antioxidant, anti-tumour, and anticarcinogenic activities (Pighin e al., 2016; Troy et al., 2016; Solarczyk et al., 2020). Different cattle breeds likely have different genetic predispositions with regard to producing CLA in the rumen. Some beef producers engage in crossbreeding programmes to improve specific meat characteristics, including CLA content (Puente et al., 2019; Solarczyk et al., 2020). Through the selective breeding of cattle, it is possible to influence the CLA content in the resulting beef (Solarczyk et al., 2020). A second important factor in determining the CLA content in raw material is cattle nutrition. Meat from pastured animals has a higher CLA content, especially if they are native breeds (Purchas et al., 2008; Litwińczuk et al., 2016). The highest contents of the C18:2 *cis*-9, *trans*-11 CLA isomer were found in beef obtained from local Polish Red bulls and ranged from 10.14 to 10.70 mg 100 ${}^{-1}$ of meat. Similarly, Litwińczuk et al. (2016) found higher percentages of CLA (0.32 %–0.43 %) and SIM (Simmental; 0.36 %–0.43 %) in the meat of bulls of three local breeds compared with in PHF (Polish Holstein–Friesian) meat (0.20 %–0.29 %).

Several sources of information suggest that humans evolved on a diet containing omega-6 and omega-3 essential fatty acids. A higher ratio of omega-6 / omega-3 fatty acids is more desirable for reducing the risk of many chronic diseases that need effective prevention. According to nutritional recommendations, the optimum omega-6 / omega-3 ratio is between 1 and 2 and should not exceed 4 (Litwińczuk et al., 2016; Momot et al., 2020). Many authors have put forward the proposal that meat obtained from cattle raised in traditional systems, where feeding is mainly based on grass foraging and haylage, has a higher percentage of polyunsaturated fatty acids and a lower omega-6 / omega-3 ratio than intensively fed animals (Randby et al., 2010; Litwińczuk et al., 2016; Pighin et al., 2016; Bermingham et al., 2021; Terry et al., 2021). In this study, the ratio of omega-3 to omega-6 acids was equal for all breeds and types of meat, with no noticeable tendency in favour of cattle of the local breed. The ratio of omega-3 to omega-6 fatty acids ranged from 2.390 in the longissimus lumborum to 4.590 in the longissimus thoracis in LM FR cattle. In other Polish studies (Momot et al., 2020), the most desirable ratio was observed in PHF × LM crosses (2.84) and in the oldest bulls (2.92).

The atherogenic index is often used to assess the nutritional value of foods (Ulbricht and Southgate, 1991; Bermingham et al., 2021). It is calculated using a ratio between saturated fatty acid (SFA) (C12:0, C14:0, and C16:0) and the sum of MUFA (monounsaturated fatty acid) and PUFA (polyunsaturated fatty acid). The atherogenic index was most favourable in the longissimus thoracis muscle in the beef cattle, regardless of the experimental group origin, at 0.726 and 0.738 for LM beef breeds and 0.741 for PRC. Bermingham et al. (2018) had a similar value in terms of the atherogenic index for ribs, roast beef, and tenderloin for various breeds of beef in New Zealand, suggesting that this type of meat has no negative impact on human health. Many studies (Litwińczuk et al., 2016; Bermingham et al., 2018; Solarczyk et al., 2020; Bermingham et al., 2021; Hammond et al., 2022) have shown that, among biologically active ingredients, the most attention is paid to the content of conjugated linoleic acid (CLA), the ratio of omega-3 to omega-6 fatty acids, and the atherogenic index (AI) in red meat. Therefore, these factors are also important when comparing the health-promoting qualities of beef.

## 5 Conclusions

In conclusion, the comparative assessment of meat composition parameters between Limousin breeds (LM FR and LM PL) and PRC unveils distinct characteristics that underscore the potential suitability of these breeds for meat production. Limousin breeds showcase higher protein content, especially evident in LM FR's substantially higher content in the longissimus lumborum, suggesting a protein-rich meat profile. Conversely, these breeds exhibit lower intramuscular fat content, aligning with preferences for leaner meat, which is notably pronounced in LM FR and, particularly, in the longissimus lumborum. The percentage differences highlight the relative changes in intramuscular fat, anserine, and carnosine content between the different breeds and muscles. Moreover, beef obtained from PRC bulls had a higher content in terms of the *cis*-9, *trans*-11 CLA isomer compared to the LM breed, which resulted in a higher nutritional potential of the meat. In turn, the lower omega-6 / omega-3 ratio in Limousin beef means a more balanced fatty acid composition, which has potential health benefits. The slightly lower atherogenicity index values calculated for LM beef indicate potential cardiovascular benefits, while the subtle differences in thrombogenicity index values suggest no difference in the associated thrombotic risk when consuming pasture-raised beef regardless of the breed origin of the meat raw material.

The mentioned studies provide valuable information to producers and consumers, highlighting the diversity of meat composition characteristics in different muscle types and the importance of adapting new breeding practices, from year-round pasture farming to market preferences. Ultimately, the results suggest that the Limousin breeds, especially LM FR, present favourable nutritional profiles in their meat, featuring higher protein content and reduced fat. However, consumer preferences, taste considerations, and specific dietary objectives should guide decisions concerning meat consumption. Regarding Polish Red cattle, their distinctive traits, such as higher intramuscular fat content, may influence meat sensory attributes. While PRC may not entirely match specialised beef breeds in all composition aspects, their attributes render them suitable for meat production. Tailoring breeding and management practices according to diverse market preferences will aid in meeting specific consumer demands. Overall, both breeds exhibit distinct characteristics, offering valuable choices in meat production and allowing producers to cater to varied consumer needs and preferences within the market.

## 6 Implications

The conclusions from the conducted research suggest that Polish Red cattle have the potential to produce meat of high nutritional value. To optimise the quality of meat obtained from local breeds, it is important to use appropriate fattening and maintenance systems while maintaining high welfare standards, the aim of which should be to obtain profitable fattening values while minimising breeding costs, such as through feeding on pastures throughout the year.

## Data Availability

The data presented in this study are available from the corresponding author upon reasonable request.
